# Relationship Between Ulnar Nerve Instability and the Degree of Ulnar Collateral Ligament Laxity in High School Baseball Pitchers

**DOI:** 10.7759/cureus.80024

**Published:** 2025-03-04

**Authors:** Yuhei Hatori, Tsuyoshi Tajika, Koichiro Yanai, Fukuhisa Ino, Ryosuke Miyamoto, Masataka Kamiyama, Tsuyoshi Sasaki, Hitoshi Shitara, Kenji Takagishi, Hirotaka Chikuda

**Affiliations:** 1 Department of Orthopaedic Surgery, Gunma University Graduate School of Medicine, Maebashi, JPN; 2 Department of Applied Rehabilitation Sciences, Graduate School of Health Sciences, Gunma University, Maebashi, JPN

**Keywords:** baseball pitcher, elbow, instability, ulnar collateral ligament, ulnar nerve, ultrasonography

## Abstract

Introduction

Ulnar nerve instability (UNI) is a common cause of ulnar neuropathy. The relationship between UNI and medial elbow instability has not yet been investigated in baseball pitchers. We investigated the association between UNI and the degree of ulnar collateral ligamentous laxity in high school baseball pitchers.

Methods

We examined 172 local high school baseball pitchers. A clinical examination assessed pitchers’ physical condition during the winter off-season from 2021 to 2023. Ultrasound examinations were conducted on the medial joint gap of both sides of the pitchers during valgus stress and non-stress conditions. The participants were divided into three groups based on the ultrasonographic findings of UNI: no instability (type N), subluxation (type S), and dislocation (type D). For the pitching side, we assessed the relationship between the type of UNI and medial elbow instability and other clinical and physical findings, including ulnar nerve symptoms, grip strength, and key pinch strength.

Results

The prevalence of UNI on the throwing side was 62% (subluxation, 32%; dislocation, 30%) and 60% (subluxation, 26%; dislocation, 34%) on the non-throwing side. Regarding the rates according to the three types of UNI, there was no significant difference between the pitching and non-pitching sides. There was a significant difference in the distance between the medial joint gap under stress and non-stress conditions, with 0.59 mm on the throwing side and 0.36 mm on the non-throwing side; however, no significant difference was found in the degree of ulnar collateral ligamentous laxity when comparing each type of UNI on the throwing side.

Conclusion

In this study of 172 high school baseball pitchers, UNI occurred on the throwing side in 62% of subjects (32% subluxation, 30% dislocation) and on the non-throwing side in 60% of subjects (26% subluxation, 34% dislocation). There was no significant difference in the rates of the three types of UNI between the pitching and non-pitching sides. Additionally, there was no association between UNI type and the presence or absence of ulnar nerve symptoms. The medial joint gap distance under both stress and non-stress conditions was significantly larger on the throwing side compared to the non-throwing side. However, no significant association was found between the different types of UNI and the degree of ulnar collateral ligamentous laxity on the throwing side in this population.

## Introduction

Ulnar nerve instability (UNI) is a condition in which the ulnar nerve slips out of the groove of the ulnar nerve during elbow flexion, causing it to dislocate or subluxate anteriorly at the medial epicondyle and potentially leading to neuritis and neuropathy [1]. However, UNI is reported to affect 11% to 56% of the general population [2-12], and previous studies suggest that it is not associated with neurological symptoms [2, 3, 5, 7]. As such, its pathological significance is currently unclear. Athletes who throw regularly often exhibit ulnar neuropathy [13]. Many studies examining UNI have focused on baseball players [14-17], and some suggest that UNI is more prevalent among pitchers than among players of other positions [14, 15, 17]. Baseball pitchers represent a specific population group of excessive and repetitive valgus stress exertion on the elbow joint. Thus, their medial collateral ligament (MCL) complex is under repetitive load, which may lead to partial or complete tear/disruption, and MCL instability may induce UNI. Although college students and major league players have been studied, research on high school baseball players is scarce, and studies specifically focused on pitchers are even rarer. Some studies suggest that UNI is more prevalent among pitchers [14, 15, 17], although no definitive consensus exists. We were unable to find any previous research on UNIs that focused on high school baseball pitchers, an age group recognized as having growth spurts and whose practice load is increasing. Recent studies indicate that professional, high school, and college-age pitchers exhibit an increase in medial joint space on the throwing side [18-22], indicating that the repetitive stress associated with throwing may alter the structure of the elbow joint. Conversely, young athletes who engage in throwing activities do not appear to experience similar changes [23]. Indeed, previous cadaver studies have shown that in the throwing motion, ulnar nerve stress at the cubital tunnel increases early in the acceleration phase at maximum elbow flexion [24] and that medial elbow instability associated with ulnar collateral ligament (UCL) dysfunction causes ulnar nerve elongation [25]; however, no studies have investigated the association between UNI and the degree of ulnar collateral ligamentous laxity in vivo. This study assessed the differences in the prevalence of UNI by hand dominance and evaluated the relationship between UNI and the degree of ulnar collateral ligamentous laxity of the throwing side in high-school baseball pitchers.

## Materials and methods

The participants were 172 male local high school baseball pitchers aged between 15 and 17 years. The inclusion criterion was male high school baseball pitchers, and the exclusion criterion was a history of trauma, such as elbow joint fracture or dislocation. During the off-seasons from 2021 to 2023, pitchers' physical condition was evaluated through clinical examinations. All participants and their guardians signed an informed consent form to participate in this study and to have their data published in a journal article, which was approved by the institutional review board of Gunma University Hospital, Maebashi, Japan (approval number 1003). This study was performed per the ethical standards laid down in the 1964 Declaration of Helsinki and its later amendments or comparable ethical standards.

Anthropometric measurements

Height was measured using a digital height meter (A&D Co. Ltd., Tokyo, Japan). Body weight was measured using a multi-frequency segmental body composition analyzer (MC780U; Tanita Corp., Tokyo, Japan).

Ultrasonographic technique

Assessment of Elbow Joint Instability

**Figure 1:**
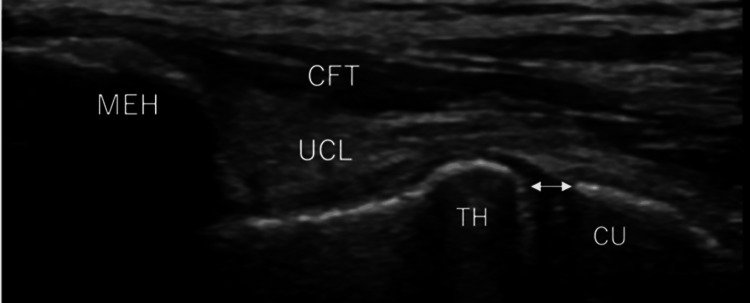
Measurement of the ulnohumeral joint width We measured the width of the ulnohumeral joint at the level of the anterior band with and without valgus gravity stress on the bilateral side. The ulnohumeral joint space was defined as the distance from the edge of the trochlea of the humerus to the edge of the coronoid process of the ulna. MEH: medial epicondyle of the humerus; TH: trochlea of the humerus; CU: coronoid process of the ulna; CFT: common flexor tendon; UCL: ulnar collateral ligament

The participants were placed on their backs with their shoulders externally rotated as much as possible. Their arm was raised to a 90° angle, their elbow was bent to 30°, and their forearm was kept in a neutral position. The medial elbow was imaged using a multifrequency 12 MHz linear-array transducer (LOGIQe; GE HealthCare; Chicago, IL). We applied gravitational stress to the forearm, which resulted in a strain on the UCL. An orthopedic surgeon with 10 years of experience in the musculoskeletal US scanned the bilateral UCLs, both with and without gravity stress, with the assistance of another orthopedic surgeon. The decision regarding the medial joint gap was made by two orthopedic surgeons, and the result of their agreement was adopted. We adopted conventional 30° elbow flexion for US evaluation of the anterior band of the UCL using the easy and verifiable gravity valgus stress method, as described in previous studies [26]. The ulnohumeral joint's width at the anterior band's level was measured without gravity valgus stress on both sides using electronic calipers (Figure 1). The ulnohumeral joint space width was defined as the distance between the edge of the trochlea of the humerus and the ulnar coronoid process of the ulna [26]. We calculated the gap between the width of the bilateral ulnohumeral joints both with and without gravity valgus stress. In our previous study [26], we established the inter-rater validity and reliability of the ulnohumeral joint space measured with electronic calipers.

Assessment of UNI

An orthopedic surgeon with 10 years of experience in the musculoskeletal US scanned the bilateral ulnar nerves with assistance from another orthopedic surgeon. After examining medial elbow instability, the elbow was maximally flexed without altering the shoulder position, and UNI was subsequently evaluated. The decision regarding the presence of UNI was made by two orthopedic surgeons, and the result of their agreement was adopted.

**Figure 2:**
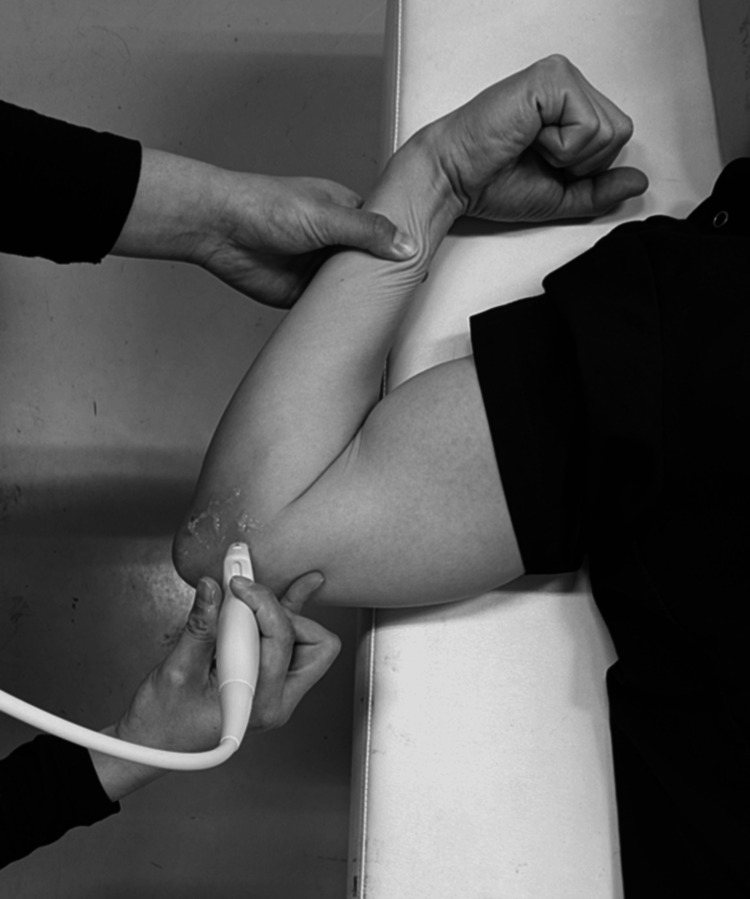
The position of the ultrasound probe during the ulnar nerve instability (UNI) evaluation

**Figure 3:**
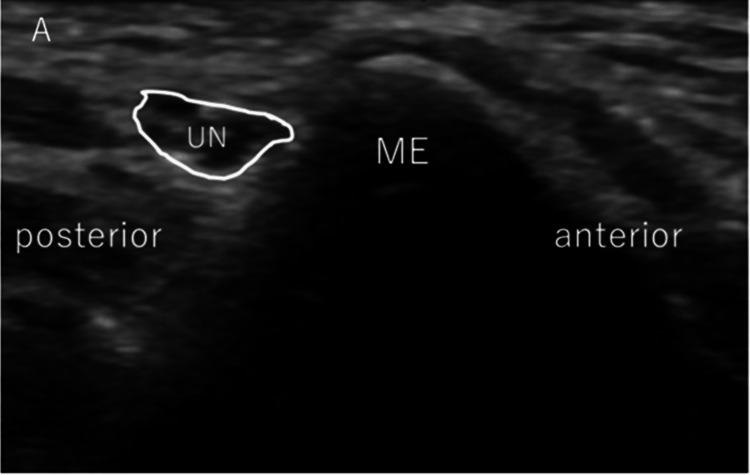
Assessment of ulnar nerve instability: normal type Transverse US images of the ulnar nerve show normal findings. ME: medial epicondyle; UN: ulnar nerve; US: ultrasonography

**Figure 4:**
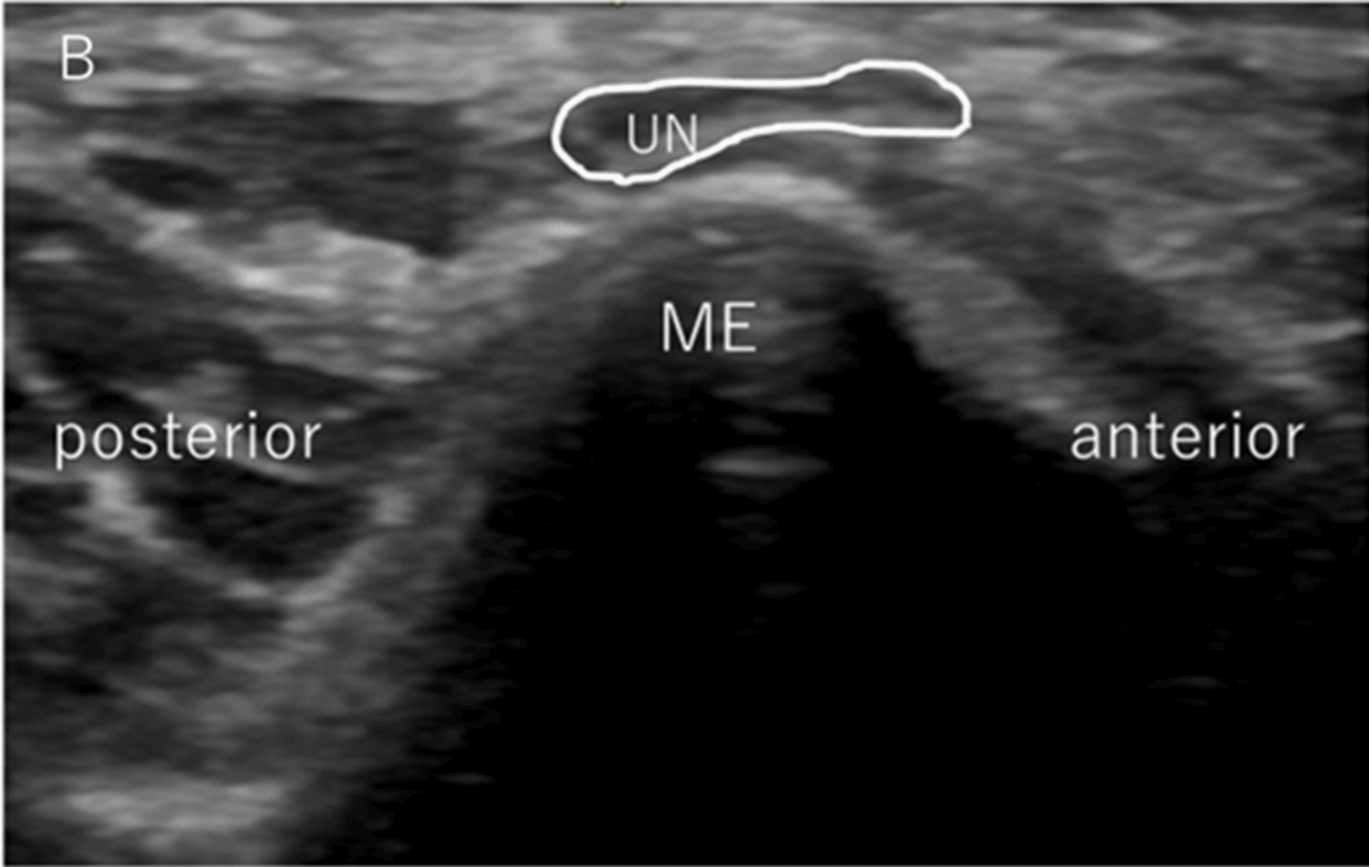
Assessment of ulnar nerve instability: subluxation type Transverse US images of the ulnar nerve show subluxation. ME: medial epicondyle; UN: ulnar nerve; US: ultrasonography

**Figure 5:**
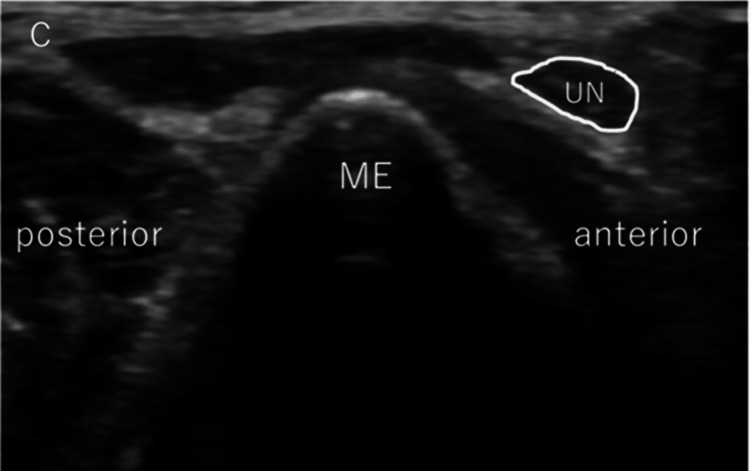
Assessment of ulnar nerve instability: dislocation type Transverse US images of the ulnar nerve show dislocation. ME: medial epicondyle; UN: ulnar nerve; US: ultrasonography

After examining medial elbow instability, we checked the movement of the ulnar nerve by passively flexing the elbow joint from 0° to the maximum flexion angle without altering the shoulder position, and UNI was subsequently evaluated (Figure 2). The maximum flexion angle of the elbow varied depending on the pitcher's body habitus, but it was always completely flexed passively. The ulnar nerve position was determined, followed by the classification reported by Okamoto et al., as follows: type N (no instability), type S (subluxation), or type D (dislocation) [11]. In type N, the ulnar nerve moves in an anteromedial direction but does not reach the tip of the epicondyle (Figure 3). In type S, subluxation occurs. The nerve is moved to the tip of the epicondyle (Figure 4). In Type D, dislocation occurs. The ulnar nerve crosses the tip of the epicondyle (Figure 5).

Subjective symptoms and muscle strength assessment

Subjective findings included asking participants whether they experienced dysesthesia in the ulnar half of the ring and little fingers. Tinel’s sign of the cubital tunnel, grip strength, and key-pinch strength were evaluated as objective findings. A digital dynamometer (Takei Scientific Instruments Co. Ltd., Tokyo, Japan) was used to measure grip strength according to the standardized position recommended by the American Society of Hand Therapists [27]. Each subject was seated shoulder adducted and in a neutral rotated position, with the elbow in approximately 90° flexion, the forearm semi-prone, and the wrist in a neutral resting position. We used a pinch gauge (MG-4320NC pinch gauge; B&L) and measured the key pinch strength according to the next procedure: the key pinch is the thumb pad to the lateral aspect of the middle phalanx of the index finger in a sitting position with the shoulder adducted and in a neutral rotated position, with the elbow in approximately 90° flexion, and the forearm and wrist in a neutral resting position. Two trials for each grip and key-pinch test were conducted to evaluate the throwing and non-throwing sides, and the average value was used for the analysis. One orthopedic surgeon conducted the tests.

Statistical analyses

After data collection, we stratified participants into three groups: type N (no ulnar nerve dislocation), type S (subluxation of the ulnar nerve), and type D (ulnar nerve dislocation) on both the throwing and non-throwing sides. Using an analysis of variance or the Kruskal-Wallis test, we compared the body mass index, grip strength, key-pinch strength, and width of the throwing-side ulnohumeral joint with and without valgus stress for each type of UNI. The differences in the presence or absence of subjective symptoms of ulnar neuropathy in the three groups were compared using a chi-square test. Data from the throwing and non-throwing sides were compared using paired t-tests. Data are shown as the mean and standard deviation. P values of <0.05 were considered to indicate statistical significance.

## Results

The prevalence of UNI on the throwing side was 62% (subluxation, 32%; dislocation, 30%) and 60% (subluxation, 26%; dislocation, 34%) on the non-throwing side. Regarding the rates according to the three types of UNI, there was no significant difference between the pitching and non-pitching sides (Table 1). The prevalence of each type of UNI in the bilateral extremities showed a similar dislocation morphology on both sides in 68% of the pitchers (Table 2). When comparing the throwing and non-throwing sides, the medial joint space was significantly larger on the throwing side under both non-gravity stress and gravity stress conditions. Additionally, the gap distance of the ulnohumeral joint with and without gravity valgus stress was significantly larger on the throwing side (Table 3). There was no significant difference between the types of UNI regarding the experience of numbness in the ring and little fingers and the presence of Tinel's sign. None of the pitchers showed numbness in the ring or little finger of the pitching hand during the medical checkup. However, eight pitchers reported numbness in their ring finger or little finger when they threw during the season (Table 4). In addition, UNI type showed no impact on grip strength, pinch strength, or BMI (Table 4). The relationship between the degree of ulnar collateral ligamentous laxity and UNI type on the pitching side is shown in Table 4. The medial joint gap distances between the stress and non-stress conditions were as follows: 0.57 mm (type N), 0.64 mm (type S), and 0.56 mm (type D). In Type D, the joint gap was significantly larger than in the other two types under non-stress and stress conditions. However, there was no difference in the distance between the medial joint gap under stress and non-stress conditions, which is used as an index of the degree of ulnar collateral ligamentous laxity, across the different types of UNI. Although we combined types S and D, both of which have UNI, and compared the two groups (Table 5), we observed no marked difference in ulnar collateral ligamentous laxity or other outcomes.

**Table 1:** Prevalence by type of ulnar nerve instability (UNI) on each side Prevalence is shown in parentheses.

Type of UNI	Throwing side (n=172)	Non‐throwing side (n=172)	Chi-square value	P-value
Type N n (%)	66 (38%)	68 (40%)	1.62	0.44
Type S n (%)	54 (32%)	44 (26%)		
Type D n (%)	52 (30%)	60 (34%)		

**Table 2:** Prevalence by type of ulnar nerve instability in the bilateral extremities Prevalence is shown in parentheses.

Laterality of ulnar nerve	Number (%)
Bilateral normal	50 (29%)
Bilateral subluxation	26 (15%)
Bilateral dislocation	41 (24%)
Unilateral subluxation	25 (15%)
Unilateral dislocation	9 (5%)
Unilateral subluxation and unilateral dislocation	21( 12%)

**Table 3:** Muscle strength and degree of ulnar collateral ligament laxity of each side Mean values are shown with the standard deviation in parentheses; Statistically significant: P < .05; ^†^Medial gap with gravity stress minus medial gap without stress

Parameter	Throwing side	Non-throwing side	t value	P-value
Grip (kg)	36.7 (6.94)	36.8 (6.64)	0.31	0.76
Key pinch (kg)	8.83 (1.55)	8.82 (1.51)	0.16	0.87
Without gravity stress (mm)	3.74 (0.63)	3.64 (0.58)	2.25	0.03
With gravity stress (mm)	4.33 (0.64)	3.98 (0.64)	8.11	<0.001
Gap distance†(mm)	0.59 (0.45)	0.36 (0.38)	5.51	<0.001

**Table 4:** The comparison of data by type of ulnar nerve instability on the throwing side BMI: body mass index Mean values are shown with the standard deviation in parentheses; Statistically significant: P < .05; ^†^Medial gap with gravity stress minus medial gap without stress

Parameter	Type N (n=66)	Type S (n=54)	Type D (n=52)	F value	P-value
BMI (kg/m²)	23.2 (3.1)	22.8 (2.6)	22.9 (2.5)	0.26	0.77
Grip (kg)	37.3 (6.4)	36.4 (7.1)	36.3 (7.5)	0.39	0.68
Key pinch (kg)	8.9 (1.5)	8.7 (1.5)	8.9 (1.6)	0.51	0.6
Medial gap of the elbow joint					
Without gravity stress (mm)	3.66 (0.65)	3.64 (0.52)	3.96 (0.65)	4.36	0.01
With gravity stress (mm)	4.22 (0.64)	4.28 (0.50)	4.51 (0.74)	3.32	0.04
Gap distance†(mm)	0.57 (0.42)	0.64 (0.43)	0.56 (0.51)	0.57	0.21
Ulnar nerve symptoms					
Dysesthesia				Chi-square value	P-value
Positive (n)	3	1	4	2.04	0.36
Negative (n)	63	53	48		
Tinel's sign					
Positive (n)	1	4	3	2.54	0.28
Negative (n)	65	50	49		

**Table 5:** The comparison of data by the presence of ulnar nerve instability on the throwing side BMI: body mass index Mean values are shown with the standard deviation in parentheses; Statistically significant: P < 0.05; ^†^Medial gap with gravity stress minus medial gap without stress.

Parameter	Type N (n=66)	Type S+Type D (n=106)	t value	P-value
BMI (kg/m²)	23.2 (3.1)	22.9 (2.5)	0.67	0.5
Grip (kg)	37.3 (6.4)	36.4 (7.3)	0.88	0.38
Key pinch (kg)	8.9 (1.5)	8.8 (1.6)	0.79	0.43
Medial gap of the elbow joint				
Without gravity stress (mm)	3.66 (0.65)	3.79 (0.60)	-1.35	0.18
With gravity stress (mm)	4.22 (0.64)	4.39 (0.64)	-1.73	0.09
Gap distance†(mm)	0.57 (0.42)	0.61 (0.47)	-0.59	0.56
Ulnar nerve symptoms				
Dysesthesia			chi-square value	P value
Positive (n)	3	5	0.003	0.96
Negative (n)	63	101		
Tinel's sign				
Positive (n)	1	7	2.37	0.12
Negative (n)	65	99		

## Discussion

This study has two main findings. First, in this study, in which the subjects were limited to pitchers, the prevalence of UNI was not significantly different between the throwing and non-throwing sides. Second, there was no association between UNI type and the degree of UCL laxity. In this study, the prevalence of UNI was high among high school baseball pitchers, but there was no significant difference between the throwing (62%) and non-throwing (60%) sides. Our study is the relative number of participants ever in a study of UNI involving only pitchers.In prior studies of non-athletes, the prevalence of UNI was variable, ranging from 11% to 56% [2-12]. The study by Cornelson et al. [6] included asymptomatic volunteers with an average age of 26 years, 56% of whom had UNI. The study by Kang et al. [9] centered on healthy college students, 55% of whom had UNI, suggesting that UNI may occur at a higher frequency in the younger generation. In this study, conducted solely on high school-aged baseball pitchers, 71% of the pitchers had UNI on one or both sides, indicating a higher prevalence relative to other studies (Table 6).

**Table 6:** The prevalence of UNI in baseball players of different ages reported in previous studies UNI: ulnar subluxation type plus ulnar dislocation type

Study	Study type	Subjects number	Subjects mean age (range)	Types of Subjects	Prevalence of UNI
Tsukada et al, (2021)	Retrospective study	Male: 60	19.6 years	College baseball players	Dominant side only: 6.7% Non-dominant side only: 3.3%; Bilateral side: 78.3%
Kabata et al, (2022)	Cross-sectional study	Male: 55; Female: 2	11.2 years (10-12 years)	Elementary school baseball players	Dominant side only: 24.6%; Non-dominant side only: 0%; Bilateral side: 11.2%
Looney et al, (2024)	Cross-sectional study	Male: 91	22 years (17-30 years)	Professional baseball pitchers	Dominant side only: 8.8%; Non-dominant side only: 3.3%; Bilateral side: 26.4%

Throwing athletes are believed to place more strain on the ulnar nerve of the throwing side; however, there are limited UNI studies focusing specifically on baseball players. Kawabata et al. [15] found that 32% of athletes between 10 and 12 years of age had UNI and that the condition was more frequently observed on the throwing side. Tai et al. [14] studied the adolescent generation and reported that UNI is more common on the throwing side and that the anterior translation distance of the ulnar nerve is significantly greater in pitchers, especially when they are in a flexed position. They speculated that ulnar nerve symptoms may not occur in athletes with UNI, suggesting that UNI could be an adaptive change in response to repeated throwing motions. A study by Looney et al. [17], involving 91 professional baseball players and focusing exclusively on pitchers, reported that the prevalence of UNI was 35.2% on the pitching side and 29.7% on the non-pitching side. Their study also concluded that UNI was not pathological, at least in their population, because ulnar nerve symptoms did not occur in the UNI group. In our study of high school pitchers, we found that UNI occurred at a relatively high frequency on both the throwing side (62% (32% subluxation, 30% dislocation)) and the nonthrowing side (60% (26% subluxation, 34% dislocation)) with no significant difference in prevalence between the two sides. This indicates a similar tendency to that observed in adults. It was thought that the side difference was reduced owing to some factors that occurred during the growth process. As in previous studies, no association was found between ulnar nerve symptoms and UNI in this study, suggesting that UNI is not necessarily a pathological condition. Tsukada et al. [16] studied college-age baseball players and reported a very high prevalence of UNI (83% (32% subluxation, 52% dislocation)), with no apparent left-right difference. They conducted a similar study of athletes in other sports, such as rugby, long-distance running, and soccer, and interestingly reported a higher incidence of UNI in rugby and baseball players. They attributed the high rate of UNI to the overuse of the upper extremities in these sports and the push-out of the ulnar nerve by the medial head of the triceps muscle. However, since the upper limb movement situation differs depending on the characteristics of the sport, the cause of UNI is thought to differ. The non-throwing arm plays an important role in baseball, both for catching and as the bottom hand while batting. As baseball players age, the time they spend playing the game increases, consequently increasing the frequency of use of the non-throwing arm as well. This may be why older baseball players may not show a difference in the frequency of UNI between the left and right sides of the upper extremities compared to young baseball players. Further detailed research is needed. There was no association between UNI type and the degree of UCL laxity. Although many studies have investigated the prevalence of UNI and the degree of medial elbow instability separately in baseball players, no previous studies have investigated these associations in high school baseball pitchers. It is known that the joint space gap in the medial elbow joint is significantly larger on the throwing side than on the nonthrowing side, regardless of the history of injury, with the throwing side showing greater valgus laxity [18-22]. A prior cadaver study showed that medial elbow instability associated with UCL dysfunction causes elongation of the ulnar nerve during the throwing motion [25]. We hypothesized that UNI may occur more frequently in pitchers with a high degree of ulnar collateral ligament laxity to protect the nerve from ulnar nerve stress due to the throwing motion. However, the medial joint gap distance between under stress and non-stress, which is the index of medial instability in this study, did not differ significantly by type of UNI. This suggests that there was no association between medial instability and UNI, at least in the high school-age group. Since the medial joint distance without gravity and valgus stress were greater in Type D, there may be an association between static elbow joint alignment and UNI, but this is not yet clear. The study by Morrow et al. reported that medial instability did not differ between the throwing and non-throwing sides in baseball players of 10-13 years of age, likely because the accumulation of valgus stress from pitching is minimal in younger players [23]. Kawabata et al. and Tai et al. reported that UNI occurs more frequently on the throwing side in this age group [14,15]. As an adaptive change to pitching, UNI may occur earlier, with medial instability potentially developing as it matures.

Our study was associated with several limitations. First, the study population was limited to high school pitchers. It should be noted that these results cannot be generalized to baseball pitchers in other age groups. Second, this study only describes the relationship between the degree of ulnar collateral ligamentous laxity and UNI in the supine position; it has not been evaluated during actual pitching motion. In future research, the relationship between the degree of ulnar collateral ligamentous laxity and dynamic changes in the ulnar nerve during each phase of the pitching motion should be investigated by simulating pitching motion in cadaveric studies. Third, we did not evaluate bone alignment. It may be helpful to add a carrying angle to the survey items and assess the relationship between bony alignment and UNI in the future. Fourth, ultrasound measurements were performed by a single physician, and the throwing and non-throwing sides were not blinded during the survey. As such, there may have been some information bias. Fifth, we performed physical examinations but did not perform electrophysiological tests on the participants. If we had been able to perform these procedures, we might have obtained data that more objectively reflected the state of the ulnar nerve. Sixth, we did not investigate the load associated with deep flexion of the elbow joint on both sides, such as the number of pitches, hits, or catches. These baseball movements are thought to be possible confounding factors in the development of UNI. We also did not evaluate the degree of triceps brachii development, which has been reported to be related to UNI in previous studies [16, 28]. In addition, this study, like previous studies on UNI, is only a cross-sectional study, which means that the causal relationship between elbow pain and ulnar neuropathy, as well as its pathophysiology, cannot be clarified. To better understand the pathogenesis of UNI and determine whether it is a risk factor for pitching disability, a prospective longitudinal study should be conducted in a younger cohort.

## Conclusions

In this study of 172 high school baseball pitchers, UNI occurred on the throwing side in 62% of the subjects (32% subluxation, 30% dislocation) and on the non-throwing side in 60% of the subjects (26% subluxation, 34% dislocation). There were no significant differences in the rates of the three types of UNI between the pitching and non-pitching sides. Baseball is a sport that involves deep flexion of both elbow joints during pitching, batting, and catching. The amount of stress on the elbow joints is also thought to increase, and these factors may be related to the similarity in UNI prevalence in both elbow joints. In addition, there was no association between the UNI type and the presence of ulnar nerve symptoms. The medial joint gap distance under both the stress and non-stress conditions was significantly larger on the throwing side than on the non-throwing side. However, no significant relationship was found between the different types of UNI and the degree of ulnar collateral ligamentous laxity on the throwing side in this population. Ulnar nerve instability may be a common symptom and may not have any pathological significance in high school baseball pitchers.
